# Combining anti-IL-7Rα antibodies with autoantigen-specific immunotherapy enhances non-specific cytokine production but fails to prevent Type 1 Diabetes

**DOI:** 10.1371/journal.pone.0214379

**Published:** 2019-03-25

**Authors:** Cristina Vazquez-Mateo, Justin Collins, Sarah J. Goldberg, Maxx Lawson, Jaileene Hernandez-Escalante, Hans Dooms

**Affiliations:** 1 Arthritis Center, Rheumatology Section, Department of Medicine, Boston University School of Medicine, Boston, Massachusetts, United States of America; 2 Department of Microbiology, Boston University School of Medicine, Boston, Massachusetts, United States of America; Children’s Hospital Boston, UNITED STATES

## Abstract

Autoantigen-specific methods to prevent and treat Type 1 Diabetes (T1D) carry high hopes to permanently cure this disease, but have largely failed in clinical trials. One suggested approach to increase the efficacy of islet antigen-specific vaccination is to combine it with a modulator of the T cell response, with the goal of reducing effector differentiation and promoting regulatory T cells (Tregs). Here we asked if addition of antibodies that block the IL-7/IL-7Rα pathway altered the T cell response to islet antigen vaccination and prevented T1D in non-obese diabetic (NOD) mice. Anti-IL-7Rα monoclonal antibodies (mAbs) reduced the numbers of islet antigen-specific T cells generated after vaccination with islet peptides and alum. However, addition of anti-IL-7Rα antibodies to peptide/alum vaccination unexpectedly increased non-specific IFN-γ, IL-2 and IL-10 cytokine production and did not result in improved prevention of T1D onset. In a second approach, we used a conjugate vaccine to deliver islet autoantigens, using Keyhole Limpet Hemocyanin (KLH) as a carrier. Islet antigen-KLH vaccination led to a significant expansion of antigen-specific Tregs and delayed diabetes onset in NOD mice. These outcomes were not further improved by addition of anti-IL-7Rα antibodies. To the contrary, blocking IL-7Rα during vaccination led to non-specific cytokine production and reduced the efficacy of a KLH-conjugated vaccine to prevent T1D. Our study thus revealed that adding anti-IL-7Rα antibodies during autoantigen immunization did not improve the efficacy of such vaccinations to prevent T1D, despite altering some aspects of the T cell response in a potentially advantageous way. Further refinement of this approach will be required to separate the beneficial from the adverse effects of anti-IL-7Rα antibodies to treat autoimmune disease.

## Introduction

Anti-IL-7Rα mAbs have shown efficacy to prevent and reverse autoimmune diabetes in NOD mice [1, 2], a widely used model of spontaneous T1D that possesses many features of the human disease [3]. Our previous studies demonstrated that IL-7Rα blockade led to increased expression of co-inhibitory receptors and reduced cytokine production in polyclonal CD4+ and CD8+ T cells [1, 2]. Moreover, blocking IL-7Rα altered the balance of Foxp3-negative naïve/effector/memory CD4+ T cells (designated hereafter as “conventional” T cells (Tconv)) and Foxp3+ regulatory CD4+ T cells (Tregs) in favor of the latter in the lymphoid organs. These changes likely contribute to the therapeutic effect of anti-IL-7Rα mAbs in preventing and reversing islet pathology. The caveat of this approach lies in the requirement for prolonged treatment [1, 2], raising concerns about instating chronic immunosuppression in patients. Hence, broadly immunosuppressive agents may be better suited for short-term administration in combination with other interventions to increase their efficacy in T1D [4].

Antigen-specific approaches have long been recognized as extremely desirable for the treatment of autoimmune diseases since it is expected they would provide a specific inhibition of the pathological immune response without causing broad immunosuppression. Many antigen-specific approaches have been effective in NOD mice [5]. In patients, various efforts are underway to deliver β-cell antigens as an immunotherapy for T1D [6, 7]. The goal of these vaccinations is to specifically target the islet-specific T cells and inhibit their pathogenic activity by inducing tolerance, inactivation or cell death. In two independent large clinical trials, the autoantigen GAD65 was used in a formulation with the adjuvant alum in order to skew the response from a pathogenic Th1 to a Th2 response and potentially expand Tregs [8–10]. Unfortunately, antigen-specific immunotherapy has so far not achieved durable prevention or remission of disease in T1D patients and, therefore, combination with an additional T cell modulator may provide a strategy to achieve positive results.

In this study, we evaluated the potential of anti-IL-7Rα mAbs to modulate the Ag-specific T cell response to vaccination with islet autoantigens in NOD mice. While addition of anti-IL-7Rα antibodies to antigen-specific immunotherapy altered parameters such as antigen-specific T cell numbers and Treg proliferation in a favorable way to potentially improve therapeutic efficacy, the combination nevertheless failed to provide increased protection against T1D in the NOD model. Surprisingly, we found that administration of anti-IL-7Rα mAbs during autoantigen immunization increased the production of bystander IL-2, IL-10 and IFN-γ and this excess cytokine production may negatively impact combination therapy. Thus, our data underscore the need to further define the optimal modalities for successfully combining anti-IL-7Rα antibodies with antigen-specific immunotherapy in T1D.

## Materials and methods

### Mice

Prediabetic female NOD mice (9–13 weeks) were purchased from The Jackson Laboratory or bred in-house. All animals were housed under specific pathogen-free conditions at Boston University Medical Campus (BUMC) on a 12 h light/dark cycle. Mice were housed in sterilized cages (≤ 5 mice/cage) with free access to water and standard mouse chow. This study was carried out in strict accordance with the recommendations in the Guide for the Care and Use of Laboratory Animals of the National Institutes of Health. The protocol (number AN-15302) was approved by the Institutional Animal Care and Use Committee (IACUC) of Boston University Medical Campus.

### Peptide-MHC tetramer detection of antigen-specific T cells

Numbers and phenotype of endogenous BDC2.5-specific CD4+ T cells for each mouse were determined in samples of pooled pancreatic and peripheral lymph nodes and spleen, using a magnetic enrichment method [11] with a PE-labeled BDC2.5 I-A^g7^ tetramer (peptide sequence: RTRPLWVRME; National Institutes of Health tetramer core facility) and anti-PE MicroBeads (Miltenyi Biotech, Germany). For flow cytometric analysis, tetramer-enriched samples were stained with antibodies for phenotypic markers, and counting beads (123count eBeads, eBioscience, USA) were added. Total numbers of tetramer-positive cells in each sample were calculated using the following formula: (event count in the tetramer+ gate/event count in the eBeads gate) x total number of eBeads per sample.

### Immunizations with alum and *in vitro* restimulation assays

For all *in vivo* experiments, mice were randomly assigned to treatment or control groups using their cage numbers. To study functional changes in islet antigen-specific T cells, mice were immunized i.p. with 10 μg of islet peptide (WE14 (from Chromogranin A) (WSRMDQLAKELTAE) or BDC2.5 mimotope (RTRPLWVRME)), mixed with Imject Alum (100 μl, administered in 100 μl of PBS) (ThermoScientific, USA) on day 0 and treated with 0.5 mg anti-IL-7Rα or IgG antibodies on days 0 and 4. On day 7, spleen and PLN cells were harvested and stained with tetramers or restimulated *in vitro* with WE14 (10 μg) and antigen-presenting cells (APCs) at a ratio of 2x10^5^ spleen or PLN cells to 4x10^5^ APCs in a flat-bottom 96-well plate for 3 days. APCs were splenocytes from untreated NOD mice, treated with 15 μg/ml of mitomycin C (Fisher Scientific, US) for 30 minutes at 37° in order to halt proliferation. FCS was reduced to 1% in the supplemented culture media to reduce non-specific proliferation. Cultures were harvested after 72 hours and analyzed by flow cytometry. For diabetes prevention studies, immunization was done with a mixture of 10 μg each of WE14, GAD(206–220) (TYEIAPVFVLLEYVT) and InsB(9–23) (SHLVEALYLVCGERG) formulated with alum.

### Conjugation of islet peptides with KLH and immunizations

Peptides are conjugated to KLH using an Imject EDC mcKLH spin kit (Thermo Fisher), closely following the manufacturer’s instructions. Briefly, equal amounts of each of four peptides (WE14 or BDC2.5 mimotope, InsB(9–23), GAD65(524–543) (SRLSKVAPVIKARMMEYGTT), and IGRP(206–214) (VYLKTNVFL)) are mixed together and conjugated at a 1:1 ratio (for example 10 mg of peptide mixture with 10 mg of KLH) with mariculture KLH (Imject mcKLH, Thermo Fisher) in Imject EDC conjugation buffer and 15% DMSO. Next, 2.5 mg of the crosslinker EDC (1-ethyl-3-[3-dimethylaminopropyl]carbodiimide hydrochloride) is added. EDC reacts with exposed carboxyl and amino groups. The reaction is incubated for 2 hours at room temperature. Next, the conjugate is purified by desalting using spin columns. The purified conjugate is sterile filtered and quantified with Nanodrop. Islet Ag-KLH conjugates (10–350 μg) are suspended in PBS and injected i.v. Anti-IL-7Rα antibodies or rat IgG (0.5 mg) are administered i.p. on the same day or 3 days later and repeated on day 4 after the initial antibody administration. PLN and spleen are harvested on day 3 days after the last mAb treatment for analysis of antigen-specific T cells with tetramer enrichment and ELISPOT.

### Antibodies and staining procedures for flow cytometry

For staining of cells directly ex vivo, single cell suspensions of PLN cells and splenocytes were prepared and red blood cells lysed using ACK lysis buffer (Gibco, USA). To distinguish live from dead cells, a Fixable Viability Dye (eBioscience, US) was used. Fc receptors were blocked with anti-CD16/CD32 antibodies for 5 minutes at 4° C before antibody staining was started. The following antibodies were used for phenotyping of murine T cells: anti-CD4 (RM4-5), anti-CD25 (PC61.5), anti-CD44 (IM7), anti-Foxp3 (FJK-16s), and anti-Ki67 (SolA15) (eBioscience, Biolegend or BD Pharmingen, US). For tetramer-stained samples, anti-CD11b (M1/70), anti-CD11c (N418) anti-B220 (RA3-6B2) antibodies (all eBioscience) were added to establish a “dump gate”, excluding irrelevant cells and reducing noise. Extracellular staining was performed by incubating with antibodies for 15–30 minutes at 4°C. All antibodies were used at a 1/100 dilution, except anti-CD44 (1/400). For intracellular staining with anti-Ki67 or -Foxp3, cells were fixed and permeabilized with a Foxp3 staining buffer set (eBioscience) according to the manufacturer’s instructions.

### Flow cytometry

Tetramer staining, phenotypic markers and cytokine production in cell populations were analyzed by multiparameter flow cytometry with an LSRII flow cytometer (BD Biosciences), and data were analyzed with FlowJo software.

### Cytokine ELISPOT assays

Briefly, 96-well MultiScreen filter plates (Merck Millipore, USA) were coated overnight at 4°C with 5 μg/ml capture antibodies for anti-IL-2, anti-IL-10 and anti-IFN-γ (MABTECH, Sweden). 1x10^6^ pooled lymph node and spleen cells were added to the wells and incubated overnight at 37°C, in the presence or absence of 0.1 μM of BDC2.5 peptide. After removal of the cells, plates were washed and 1 μg/ml biotinylated anti-IL-2, anti-IL-10 and anti-IFN-γ detection antibodies (MABTECH) added for 1 hour at room temperature. Plates were washed and spots detected by incubation with streptavidin alkaline phosphatase (Jackson ImmunoResearch, USA), followed by Vector Blue AP substrate (Vector Laboratories, USA). Number of spots was calculated with an ImmunoSpot analyzer and software (ImmunoSpot, USA).

### Diabetes assessment

Diabetes incidence was followed weekly by urine analysis with Diastix strips and measuring of blood glucose levels with a Contour glucose meter (both Bayer, USA). The percentage of diabetic mice (glucose levels >250mg/dL) was calculated using the Kaplan-Meier survival curves method.

### Histology

Histological analysis was performed by fixing pancreata in 10% (vol/vol) buffered formalin and staining 5-μm paraffin-embedded tissue sections with hematoxylin & eosin (H&E); 10 sections per pancreas were blindly scored for insulitis using an Olympus BH-2 microscope. Scores are as follows: 0 = no infiltrate, 1 = 0–25%, 2 = 25–75%, 3 = > 75%. Images were captured with a Micropublisher 5.0 RTV digital camera (Q Imaging).

### Statistics

Statistically significant differences between groups were determined with one- or two-tailed unpaired or paired t tests or with one-way ANOVA with Bonferroni’s multiple comparison test (ELISPOT assays), using Graph Pad Prism. P values≤0.05 were considered significant. Horizontal lines in graphs indicate statistical significance (* = p≤0.05, ** = p≤0.005, *** = p≤0.0005, ns = p>0.05). For diabetes incidence studies, significance was determined using the Mantel-Cox log-rank test.

## Results

### Combining alum-formulated islet autoantigen immunization with anti-IL-7Rα mAbs reduces islet antigen-specific T cells but enhances non-specific cytokine production

Alum has been used as an adjuvant in two large clinical trials attempting to vaccinate recent-onset T1D patients with GAD65 [12, 13]. These trials showed only temporary efficacy at stabilizing C-peptide levels, underscoring the need to investigate whether combination with an additional immune modulator had the capacity to robustly modulate the induced anti-islet T cell response in favor of reduced effector functions and increased regulation.

**Fig 1:**
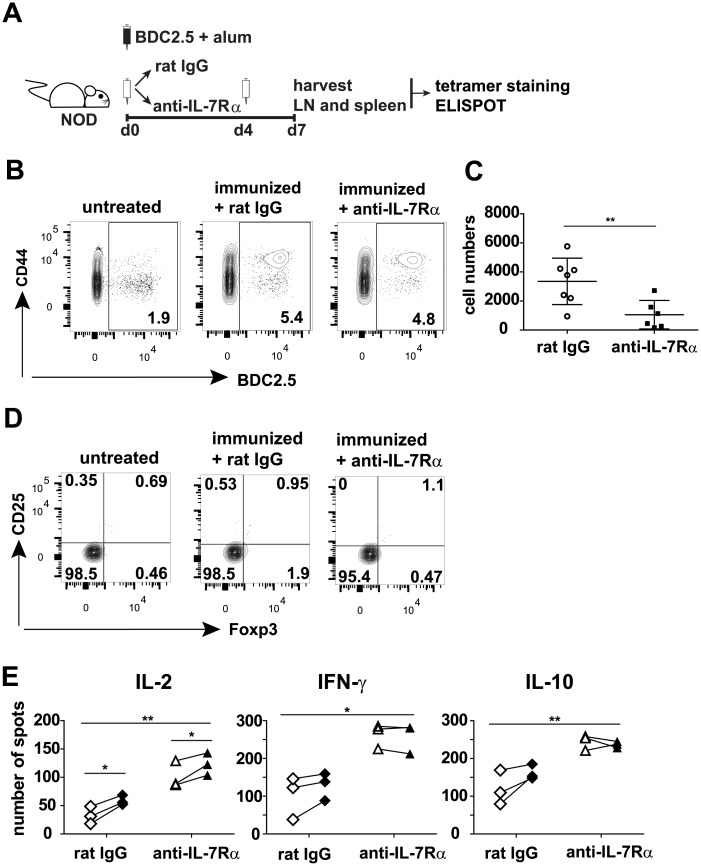
Immunization with islet antigen and alum does not expand autoantigen-specific Tregs, even in the presence of anti-IL-7Rα antibodies. (A) Experimental design outline: 9-week old female prediabetic NOD mice were immunized with alum-formulated BDC2.5 peptide mimotope (10 μg) and treated with anti-IL-7Rα or rat IgG antibodies (0.5 mg) on days 0 and 4. On day 7, pancreatic and peripheral lymph nodes and spleen were harvested and, after magnetic enrichment of BDC2.5 tetramer+ cells, numbers and phenotype of endogenous BDC2.5+ T cells were determined by flow cytometry. BDC2.5 antigen-specific cytokine-producing cells were quantified with ELISPOT assays. (B) Representative dot plots showing CD44 expression on BDC2.5 tetramer+ cells and bystander polyclonal CD4+ T cells. (C) Absolute numbers of BDC2.5+ T cells present in individual mice from three separate experiments ± SD (n = 6 or 7). (D) Representative dot plots showing Foxp3 and CD25 expression within the BDC2.5 tetramer+ T cell gate. (E) Graphs show number of spots, representing cells producing IL-2, IFN-γ or IL-10, as determined by ELISPOT assays with cells from immunized mice, without (open symbols) or with (closed symbols) adding BDC2.5 peptide to the in vitro cultures (n = 3). Unpaired t-test (C) and ANOVA with Bonferroni’s posttest (E) were used for statistical analysis, ** = p≤0.005, *** = p≤0.0005.

We immunized pre-diabetic NOD mice with BDC2.5 peptide and alum in combination with anti-IL-7Rα mAbs or rat IgG as a control (Fig 1A). To analyze the impact of anti-IL-7Rα blockade on the Ag-specific T cells responding to vaccination in vivo, lymph nodes and spleen were harvested on day 7 after immunization, and magnetic enrichment with a BDC2.5 Class II tetramer was used to detect and enumerate endogenous BDC2.5-specific T cells directly ex vivo (for gating strategy, see S1 Fig). Fig 1B shows that a large proportion of tetramer+ BDC2.5 T cells isolated from lymph nodes and spleens of immunized animals had increased CD44 expression, indicating that these cells were activated in response to immunization. Blocking IL-7Rα during immunization did not inhibit this activation (Fig 1B) but reduced the total number of recovered BDC2.5 T cells (Fig 1C). BDC2.5 T cells activated by immunization with BDC2.5 peptide and alum did contain very few Foxp3+ Tregs, both in the presence of rat IgG or anti-IL-7Rα antibodies (Fig 1D). This finding is contrary to what has been suggested, without direct evidence, in other human and mouse studies using formulations of autoantigens and alum [10, 14]. Finally, we decided to assess the impact of adding anti-IL-7Rα mAbs during immunization on effector function of the stimulated BDC2.5+ T cells, using an ELISPOT assay. Surprisingly, adding anti-IL-7Rα mAbs during priming *in vivo* resulted in increased IL-2, IFN-γ and IL-10 production (Fig 1E). The low number of remaining BDC2.5+ T cells (Fig 1C) after anti-IL-7Rα treatment and the limited induction of cytokines in the presence of cognate peptide, strongly indicate that bystander, non-antigen-specific immune cells are the source of enhanced cytokine secretion after IL-7Rα blockade.

**Fig 2:**
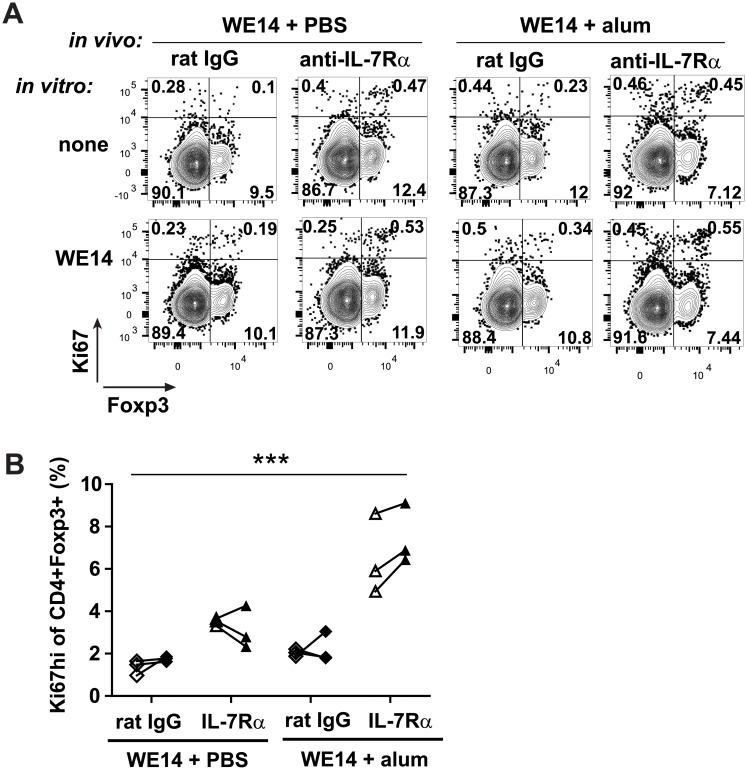
Combining IL-7Rα blockade with autoantigen and alum immunization promotes Treg proliferation in restimulation cultures. 9-week old female prediabetic NOD mice were immunized with islet peptide WE14 and alum or PBS and treated with anti-IL-7Rα mAbs or rat IgG antibodies on days 0 and 4 as in Fig 1A. On day 7, spleen and PLN cells were harvested and restimulated *in vitro* with APCs with or without added WE14. Cultures were harvested after 72 hrs and analyzed by flow cytometry. (A) Representative dot plots show percentages of proliferating (Ki67^high^), activated (CD44^high^) cells gated on CD4^+^Foxp3^+^Tregs from the PLN. (B) Graphs show proliferating Tregs in the absence (white symbols) or presence (black symbols) of WE14 in cultures from PLN (left) and spleen (right). Each symbol represents an individual mouse (n = 3). t-tests (unpaired or paired) were used for statistical analysis, * = p≤0.05, ** = p≤0.005, *** = p≤0.0005.

To further evaluate the impact of anti-IL-7Rα mAbs on the T cell response generated after immunization with islet autoantigen and alum, pancreatic lymph nodes (PLN) and spleens were harvested seven days after vaccination with alum-formulated WE14 peptide (the natural ligand of BDC2.5 T cells [15]) and cells were stimulated *in vitro* with WE14 and mitomycin C-treated splenic APCs. Proliferation of CD4+ Tconv vs Tregs in these cultures was analyzed 72 hours later by staining cells for Ki67, a marker of proliferating cells, and Foxp3, followed by flow cytometry (Fig 2A). Strikingly, we consistently detected a larger population of Ki67^hi^Foxp3+ Tregs in cultures from anti-IL-7Rα-treated animals, regardless of the presence of alum during antigen delivery (Fig 2B). Adding WE14 peptide to *in vitro* cultures did not significantly increase proliferation (Fig 2B). Together with the findings from the ELISPOT assays (Fig 1E), these experiments suggest that increased IL-2 production by bystander T cells after IL-7Rα blockade promoted the proliferation of polyclonal Tregs in these cultures.

### Combining anti-IL-7Rα mAbs with autoantigen/alum vaccination failed to prevent T1D in NOD mice

**Fig 3:**
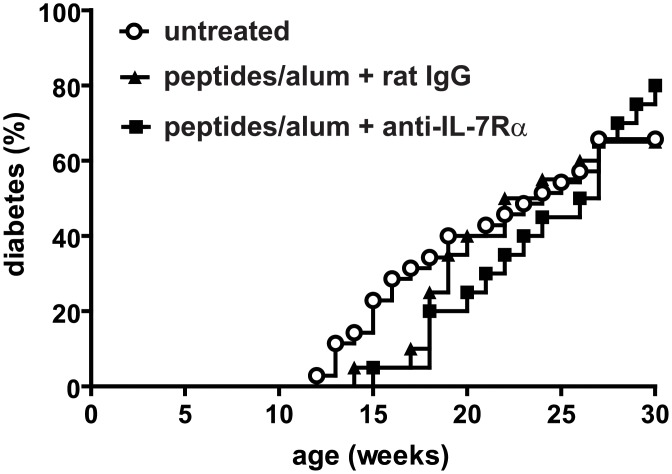
Combining anti-IL-7Rα antibodies with autoantigen and alum vaccination does not significantly delay T1D onset. 11-week old female NOD mice were i.p. injected with a mixture of three islet peptides (InsB:9–23, GAD65:206–220 and WE14, 10 μg each) formulated in alum, and rat IgG or anti-IL-7Rα antibodies (0.5 mg). On day 4, an additional dose of antibodies was administered. Mice were followed until 30 weeks of age for diabetes onset. Kaplan-Meier plot shows percentage diabetic mice in function of age within each treated group (n = 20), as well as incidence in our untreated NOD colony (n = 35). Results are pooled from two independent experiments.

Finally, we assessed whether addition of anti-IL-7Rα mAbs during vaccination with islet autoantigens and alum would improve the efficacy of this approach in secondary prevention of T1D. Cohorts of 11-week old female NOD mice (n = 10) were immunized i.p. with a mixture of the islet peptides InsB(9–23), GAD65(206–220) and WE14, formulated with alum; anti-IL-7Rα or rat IgG antibodies were administered on days 0 and 4. Diabetes incidence was followed for 30 weeks. Although vaccination initially appeared to delay the earliest age of diabetes onset by 2–3 weeks, compared to what is typical in our colony (~12 weeks), at 30 weeks of age there was no statistically significant difference in incidence between the groups (Fig 3). Hence, these data confirmed the findings by Dr. von Herrath et al. that GAD-alum alone does not protect mice from diabetes [14], and that reducing the responding islet-specific T cells with anti-IL-7Rα mAbs (Fig 1C) was not sufficient to alter the outcome of this vaccination strategy.

### Immunization with islet antigen-KLH conjugates expands antigen-specific Tregs independently of anti-IL-7Rα antibody addition

**Fig 4:**
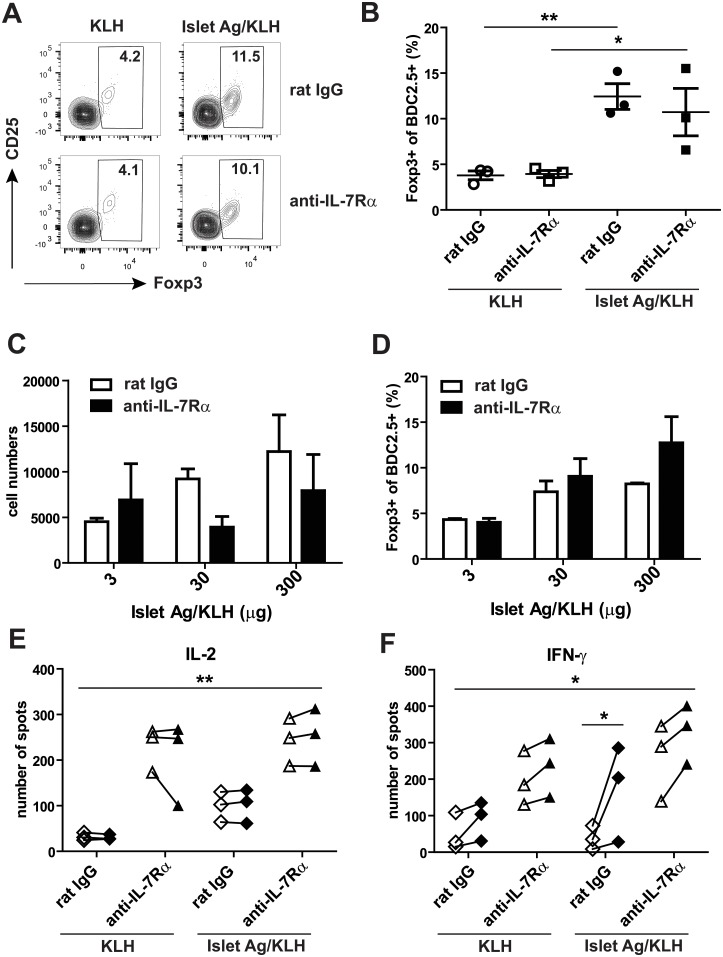
Anti-IL-7Rα mAbs promote cytokine production but not antigen-specific Tregs after islet Ag-KLH immunization. 11–13 week old female prediabetic NOD mice were immunized with islet Ag-KLH or KLH in combination with anti-IL-7Rα antibodies or rat IgG (0.5 mg) administered on days 0 and 4. PLN and spleens were harvested on day 7 or 8. BDC2.5-specific T cells were enriched and identified with tetramers as in Fig 1. (A) 350 μg of islet Ag-KLH and 175 μg of KLH were used for the immunization. Contour plots are gated on CD4+BDC2.5+ T cells and show Foxp3 and CD25 expression within this population. The percentages of Foxp3+ Tregs within the BDC2.5+ T cells on day 8 are indicated. (B) Graph shows a summary of the percentages of Foxp3+ Tregs within the BDC2.5+ T cell population, as gated in A (n = 3). (C) Mice were immunized with 300, 30 or 3 μg of islet Ag-KLH and bar graph shows absolute numbers of BDC2.5+ T cells present in individual mice on day 7 ± SD (n = 3). (D) Bar graph shows the percentages of Foxp3+ Tregs within the BDC2.5+ T cells, as in B. (E, F) Mice were immunized with 350 μg of islet Ag-KLH or 175 μg of KLH and, on day 7, 1 x 10^6^ splenocytes were used to determine cytokine-producing cells. Graphs show number of spots, representing cells producing IL-2 (E) and IFN-γ (F) as determined by ELISPOT assays with (closed symbols) or without (open symbols) adding a mixture of the islet Ags used for immunization to the in vitro cultures (n = 3). ANOVA with Bonferroni’s post test were used for statistical analysis, ** = p≤0.005, *** = p≤0.0005.

KLH is a large, strongly antigenic protein that is used as a carrier for peptide antigens in order to elicit strong immune responses against these peptides during vaccination. Here, we asked whether the response to immunization with islet autoantigens conjugated to KLH could be skewed towards a more regulatory phenotype through addition of anti-IL-7Rα mAbs. In a first experiment, equal amounts of islet Ags (BDC2.5 mimotope, GAD65(524–543), InsB(9–23) and IGRP(206–214)) were conjugated to KLH and pre-diabetic NOD mice were injected with these islet Ag-KLH conjugates (350 μg) or unconjugated KLH (175 μg) i.v. in the absence or presence of anti-IL-7Rα mAbs or rat IgG. PLN and spleens were harvested on day 8 and endogenous antigen-specific BDC2.5+ T cells visualized by magnetic enrichment and staining with BDC2.5 tetramers. The presence of Foxp3+ Tregs within the BDC2.5 cells was determined and, interestingly, we found that islet Ag-KLH led to a significant expansion of antigen-specific Tregs (up to 15% of the population) (Fig 4A and 4B), contrary to immunization with alum. However, Treg expansion was not further enhanced by anti-IL-7Rα mAbs. Next, we performed a dose response experiment and found that anti-IL-7Rα mAbs appeared to modestly reduce antigen-specific T cell numbers (Fig 4C) generated with 30 and 300 μg of islet Ag-KLH conjugates but no statistically significant differences were observed in Treg frequencies (Fig 4D). Finally, IL-2 and IFN-γ production were determined by ELISPOT analysis and, similarly to immunization with alum, administration of anti-IL-7Rα mAbs significantly enhanced cytokine production, independently of the presence of autoantigen during immunization (Fig 4E and 4F). Addition of the peptides used for immunization revealed the presence of some antigen-dependent IFN-γ-producing cells (Fig 4F). Thus, these experiments confirmed that anti-IL-7Rα mAbs potently enhance bystander, non-specific cytokine production when co-administered during peptide antigen immunization.

### Vaccination with conjugated islet Ag-KLH delays T1D onset in NOD mice

**Fig 5:**
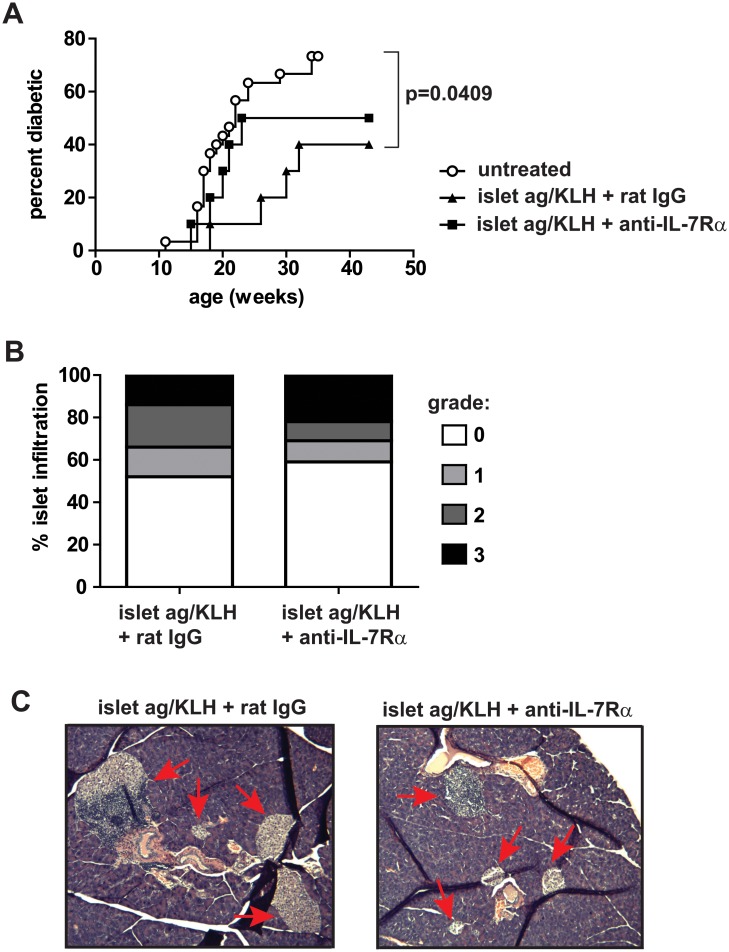
Vaccination with islet Ag-KLH delays T1D onset in NOD mice. 9-week old female NOD mice were injected with 100 μg islet Ag-KLH (i.v.) and, on day 4 and 8, 0.5 mg rat IgG or anti-IL-7Rα antibodies (i.p.). Mice were followed until 43 weeks of age for diabetes onset. (A) Kaplan-Meier plot shows percentage diabetic mice in function of age within each treated group (n = 10), as well as incidence in our untreated NOD colony (n = 30). (B) Infiltration of pancreatic islets from treated NOD mice from A that were not diabetic at 43 weeks, quantified as percentages of islets showing the indicated histological scores determined after H&E staining (rat IgG, n = 6; anti-IL-7Rα, n = 5). (C) Representative images of islets (indicated with red arrows) from pancreata of NOD mice analyzed in B.

Since immunization with islet Ag-KLH conjugates led to the generation of antigen-specific Tregs, we decided to evaluate whether this vaccination method carried therapeutic potential for T1D. Two cohorts of NOD mice were injected with islet Ag-KLH and anti-IL-7Rα mAbs (n = 10) or rat IgG (n = 10), and T1D incidence followed for 34 weeks. We found that islet Ag-KLH significantly delayed and in some cases prevented T1D onset (Fig 5A), when compared to the overall incidence in untreated animals in our colony. However, addition of anti-IL-7Rα mAbs precluded this protective effect, perhaps due to excessive pro-inflammatory cytokine production. Histological analysis of islet infiltration in the protected animals of both treatment groups revealed no major differences in overall numbers of insulitic islets, albeit that mice receiving anti-IL-7Rα mAbs showed a trend towards more severe immune cell infiltration per islet (Fig 5B and 5C). Hence, these results warrant further exploration of KLH-conjugated autoantigens as a vaccination method to induce Tregs and protect against autoimmunity. At the same time, the outcome cautions against the use of anti-IL-7Rα mAbs in combination therapy.

## Discussion

Our study explored the feasibility of using anti-IL-7Rα mAbs in combination with an islet antigen vaccination method to modulate an autoantigen-specific T cell response and prevent T1D. The rationale was based on our previous data showing that anti-IL-7Rα mAbs altered the balance of polyclonal Tregs/Tconv cells in favor of Tregs [1] and, if administered for a prolonged period of time, prevented and reversed T1D in NOD mice. We reasoned that short-term administration of anti-IL-7Rα mAbs during antigen-specific immunotherapy might promote the generation of islet antigen-specific Tregs while reducing the survival of activated effector and memory T cells. In such a scenario the immunomodulatory activity of anti-IL-7Rα mAbs could be exploited while avoiding long-term side effects such as lymphopenia and immunodeficiency. While the observed reduction in antigen-specific T cell numbers after immunization with peptide plus alum and anti-IL-7Rα mAbs was predictable, this was not accompanied by an increase in antigen-specific Tregs or T1D prevention. Immunization with islet peptides conjugated to KLH did expand antigen-specific Tregs but addition of anti-IL-7Rα mAbs did not further increase the yield or improve protection. Moreover, we unexpectedly found that anti-IL-7Rα mAbs significantly increased bystander cytokine production, potentially compromising its use in combination with vaccination against T1D.

Immunotherapies for T1D can largely be divided in two categories: non-specific immunosuppression and interventions that target islet antigen-specific T cells (for an overview see [16, 17]). Some success in preserving C-peptide secretion in patients has been achieved with non-specific immunomodulatory drugs targeting polyclonal T cells (e.g. teplizumab [18], alefacept [19]) or B cells (e.g. rituximab [20]). However, these have not led to long-term restoration of normoglycemia. Novel approaches to target polyclonal T and B cells show promise in NOD mice, e.g. anti-CD22 antibodies coupled to calicheamicin was effective at depleting pancreatic B cells [21]. It will be important to design such strategies in a way that regulatory T and B cell populations are preserved [22]. Finally, developing immunotherapeutic methods to protect islet allograft transplants also remains an important avenue of research carrying the potential to help fully restore islet function [23–25].

Therapies for T1D targeted at islet antigen-specific T cells could avoid systemic toxicity and immunosuppression caused by broad immunosuppressants [26]. Two fundamentally different approaches have been tried: those in which peptide or protein is administered as an aqueous solution aimed at tolerizing T cells vs those in which an adjuvant is added, aimed at activating the T cells in way that modulates their effector function, for example by generating increased Tregs or shifting the Th1/Th2 balance [6, 27]. Although promising in NOD mice, clinical trials focused on antigen-specific (insulin, GAD65) immunosuppression have failed to preserve insulin production in T1D patients, presumably because long-lasting tolerance to islet antigens was not induced [12, 13, 26]. It has therefore been proposed that combination with an immunomodulatory agent may improve the performance of antigen-specific immunotherapies for T1D and other autoimmune diseases [4]. Based on our previous studies, we identified anti-IL-7Rα mAbs as an interesting candidate for combination therapy: IL-7Rα blockade prevents and reverses T1D by targeting IL-7-dependent effector/memory T cells but long-term administration is required to achieve this effect [1, 2, 28, 29]. Such chronic treatment raised concerns about immunosuppression and increased susceptibility to infections. Therefore, we reasoned that short-term addition of anti-IL-7Rα mAbs during immunization with islet autoantigens may be sufficient to primarily target the primed T cells for therapeutic immunomodulation. Importantly, Tregs express low levels of IL-7Rα [30] and, in the lymphoid organs, are not thought to be strongly dependent on the cytokine. This differential IL-7Rα expression and function in effector/memory T cells and Tregs thus suggested that blocking IL-7Rα during and following vaccination would preferentially reduce effector/memory T cells while preserving Tregs, a desirable outcome for autoimmune therapy. Using tetramers to analyze the antigen-specific T cell response after islet peptide and alum immunization, we did not observe a significant number of Tregs within the BDC2.5+ T cell population, nor did this subset increase with anti-IL-7Rα blockade. At first, these data appear at odds with vaccination studies using GAD-alum in humans and mice, which have consistently shown an increase in the Foxp3+ Treg population [10, 14, 31]. However, these studies do not show direct evidence by tetramer staining that these Tregs are antigen-specific [10, 14, 31]. To the contrary, a recent report shows that in vitro expanded GAD65-specific T cells from GAD-alum-treated patients did not contain increased proportions of Tregs [32], further underscoring the difficulty to preferentially expand antigen-specific Tregs with alum. Moreover, Tregs from GAD65-alum immunized patients did not possess enhanced suppressive function [32]. Together, these and our studies suggest that increases in Foxp3+ Treg responses documented after in vitro restimulation with antigen are caused by an indirect effect on polyclonal Tregs. Non-specific IL-2 production in T cells, acting on bystander Tregs, is a candidate mechanism for this effect [33].

Increasing the numbers and function of Tregs is currently actively being pursued as a therapeutic strategy for T1D [34]. The rationale for this approach is based on evidence that Tregs are reduced and/or defective in T1D patients and NOD mice [35, 36], possibly due to defects in the IL-2/IL-2Rα pathway [36–40]. Based on these findings, preclinical studies in NOD mice have demonstrated therapeutic efficacy of low doses of IL-2 [36] and clinical trials have been initiated with this cytokine [41]. Our results demonstrating that anti-IL-7Rα treatment induced proliferation in the polyclonal Treg population suggests that IL-7 signaling negatively regulates IL-2 production in effector/memory T cells, limiting IL-2 availability for polyclonal Tregs. It remains surprising however that islet autoantigen-specific Tregs did not respond in the same way, since no increase in their frequency was observed within the tetramer+ populations after immunization with alum or KLH as the adjuvant in combination with anti-IL-7Rα mAbs. Interestingly, it was recently reported that islet autoantigen-specific Tregs express lower levels of CD25 as compared to polyclonal populations, which may explain their reduced responsiveness to IL-2 [42]. One particularly intriguing finding from our study is that using KLH as a carrier protein for autoantigens does promote expansion of antigen-specific Tregs within the tetramer+ population and may represent a promising delivery method for autoantigen vaccination. Addition of anti-IL-7Rα mAbs however worsened the preventive capacity of islet Ag-KLH vaccination, despite similar Treg expansion. It is feasible that excessive production of IFN-γ and other pro-inflammatory cytokines induced by IL-7Rα blockade undercut the protective activity of immunization with islet Ag-KLH. It is also important to take into account that the role of IL-7 in Treg biology remains controversial and both positive and negative functions have been reported [43, 44].

Finally, it is relevant to note here that anti-IL-7Rα mAbs are being tested in the clinic for autoimmune diseases (NCT01808482, NCT03239600, NCT01740609, NCT02045732, NCT02038764). Our finding that under certain circumstances use of these antibodies results in excessive cytokine production should be considered as a potential safety issue. While we and others previously documented that longer-term treatment with anti-IL-7Rα mAbs alone resulted in a reduction in cytokine-producing CD4+ T cells [1, 45], it appears that this may be preceded by a burst of cytokine production early on, followed perhaps by deletion of these highly activated T cells. Thus, this finding has important consequences for further clinical development of this target.

In conclusion, our study shows that blocking IL-7Rα has the capacity to modulate the T cell response to vaccination, but significant work remains to be done to determine whether an optimal combination of antigens, adjuvant and antibody, and timing of administration, can be found to avoid negative side effects and successfully use anti-IL-7Rα mAbs in combination immunotherapy.

## Supporting information

S1 Fig Representative gating strategy to identify antigen-specific CD4+ T cells with BDC2.5 pMHC II/PE tetramers. (PDF)
