# Supplementary material for: Combining anti-IL-7Rα antibodies with autoantigen-specific immunotherapy enhances non-specific cytokine production but fails to prevent Type 1 Diabetes
Source: PLoS One. 2019 Mar 25;14(3):e0214379. doi: 10.1371/journal.pone.0214379 (PMC6433345; doi:10.1371/journal.pone.0214379)
Supplement: S1 Fig — (PDF) [file pone.0214379.s001.pdf]

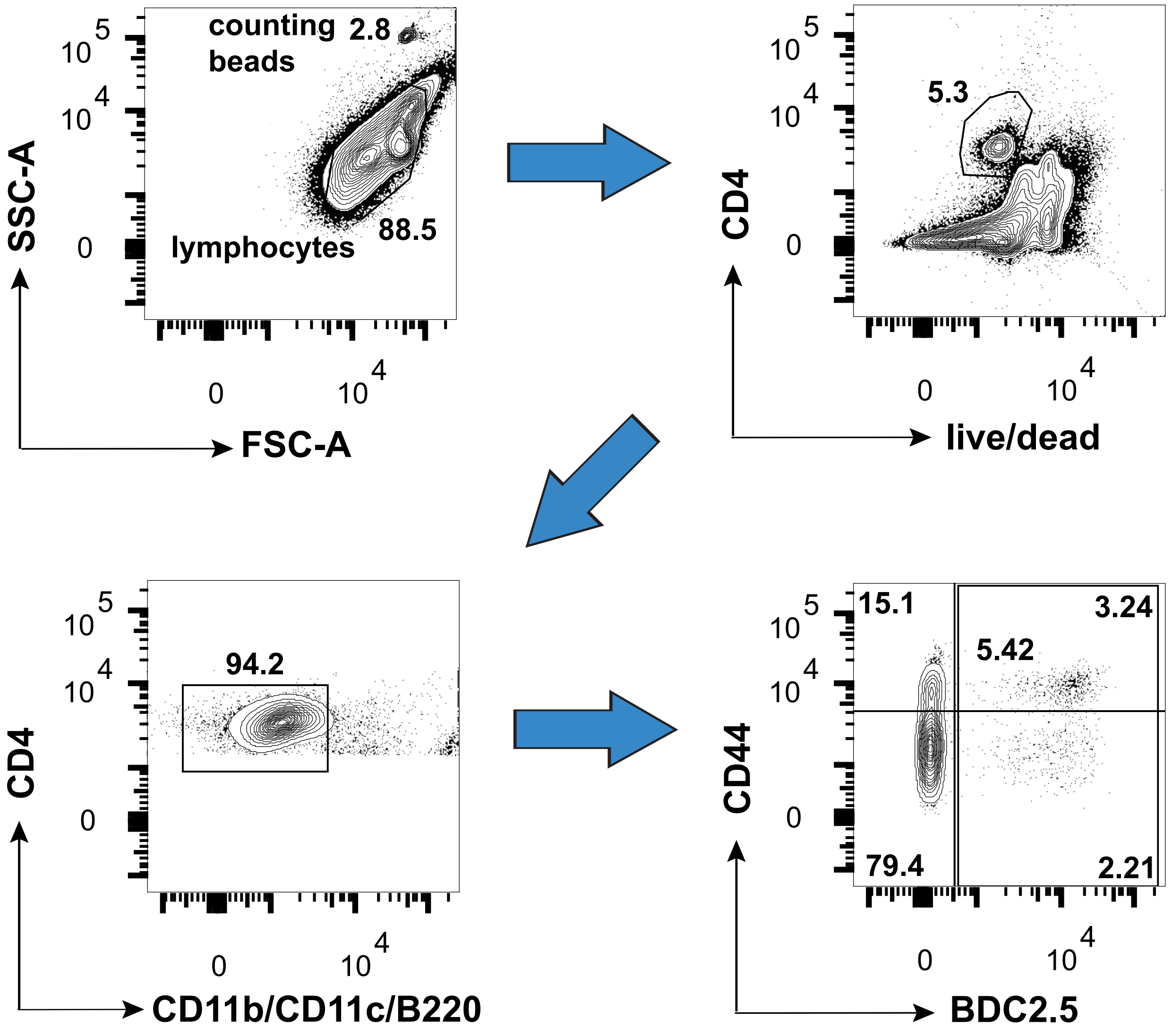

S1 Figure: Representative gating strategy to identify antigen-specific CD4+ T cells with BDC2.5 pMHC II/PE tetramers
